# The effects of spaceflight and fracture healing on distant skeletal sites

**DOI:** 10.1038/s41598-019-47695-3

**Published:** 2019-08-06

**Authors:** Ushashi C. Dadwal, Kevin A. Maupin, Ariane Zamarioli, Aamir Tucker, Jonathan S. Harris, James P. Fischer, Jeffery D. Rytlewski, David C. Scofield, Austin E. Wininger, Fazal Ur Rehman Bhatti, Marta Alvarez, Paul J. Childress, Nabarun Chakraborty, Aarti Gautam, Rasha Hammamieh, Melissa A. Kacena

**Affiliations:** 1 Department of Orthopaedic Surgery, Indiana University School of Medicine, Indianapolis, IN USA; 2 Richard L. Roudebush VA Medical Center, Indianapolis, IN USA; 3 Ribeirão Preto Medical School, Ribeirão Preto, SP Brazil; 4 U.S. Army Center for Environmental Health Research, Fort Detrick, MD USA; 5 Geneva Foundation, Fort Detrick, MD USA

**Keywords:** Bone, Bone quality and biomechanics

## Abstract

Spaceflight results in reduced mechanical loading of the skeleton, which leads to dramatic bone loss. Low bone mass is associated with increased fracture risk, and this combination may compromise future, long-term, spaceflight missions. Here, we examined the systemic effects of spaceflight and fracture surgery/healing on several non-injured bones within the axial and appendicular skeleton. Forty C57BL/6, male mice were randomized into the following groups: (1) Sham surgery mice housed on the earth (Ground + Sham); (2) Femoral segmental bone defect surgery mice housed on the earth (Ground + Surgery); (3) Sham surgery mice housed in spaceflight (Flight + Sham); and (4) Femoral segmental bone defect surgery mice housed in spaceflight (Flight + Surgery). Mice were 9 weeks old at the time of launch and were euthanized approximately 4 weeks after launch. Micro-computed tomography (μCT) was used to evaluate standard bone parameters in the tibia, humerus, sternebra, vertebrae, ribs, calvarium, mandible, and incisor. One intriguing finding was that both spaceflight and surgery resulted in virtually identical losses in tibial trabecular bone volume fraction, BV/TV (24–28% reduction). Another important finding was that surgery markedly changed tibial cortical bone geometry. Understanding how spaceflight, surgery, and their combination impact non-injured bones will improve treatment strategies for astronauts and terrestrial humans alike.

## Introduction

International space programs have received more attention and funding in recent years^1–3^, including exploration of extraterrestrial bodies like the moon and Mars. This spike in interest will involve greater human participation; therefore, several well-documented consequences of space travel, including substantial muscle and skeleton loss^4–6^, will also need to be mitigated. During International Space Station (ISS) missions, seven of eight cosmonauts experienced a reduction in bone mineral density (BMD) (2.5–10.6%) in the lumbar vertebrae, all eight showed decreased BMD in the femur (3–10%), and four of the eight showed a 1.7–10.5% decrease in BMD in the femoral neck^7^. Early studies demonstrated that exposure to the microgravity environment of space resulted in losses in the spine, femoral neck, trochanter, and pelvis of about 1%–1.6%, with considerable variation between individuals^8^. More recently, a comprehensive study on identical twins (astronaut Scott Kelly and his brother, Mark Kelly) demonstrated that spaceflight causes significant changes in gene expression (particularly after 6 months of spaceflight exposure), epigenetic signatures, vascular remodeling, and inflammation^9^. Because spaceflight profoundly alters physiology and bone mass, fracture healing could possibly also be impacted. Indeed, one consequence of severe BMD loss is fracture risk. Astronauts spending 6 months in spaceflight have on average a 10% loss in BMD, which is 10-fold greater than the BMD loss observed in post-menopausal women^10,11^. Indeed, predictive models^12,13^ suggest the likelihood of wrist, spinal, and hip fractures increasing in astronauts due to compromised bone strength; however, the systemic response to fracture injury and repair on other bones is not well known.

This study is part of a larger project designed to investigate the response to fracture injuries in space and fracture healing therapeutics. As plans for long-term human colonization become a reality, patients with poor bone health due to both reduced mechanical loading and microgravity will require comprehensive treatment. This manuscript, therefore, is intended to characterize the systemic effects of fracture healing and of spaceflight, separately and combined, on both the axial and appendicular skeleton via micro-computed tomography (µCT) analyses.

## Results

**Figure 1:**
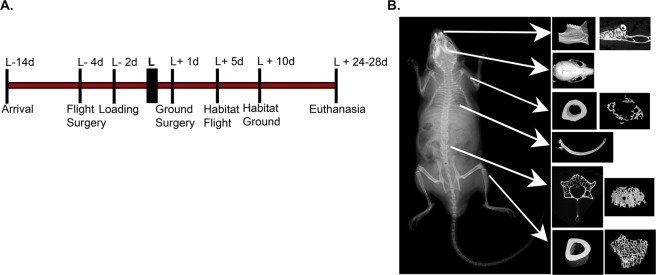
Experimental design and timeline. (**A**) Timeline detailing the overall experimental design including launch preparation/mouse acclimation, launch, and mouse euthanasia. (**B**) X-ray image of mouse skeleton with white arrows indicating the bones that were collected/analyzed. Representative reconstructed µCT images for each of these bones are shown.

In these studies, a segmental bone defect surgery was performed on the right femur in half of the mice (Surgery). The other half of the mice served as unoperated control mice (Sham). Here we sought to examine the systemic impacts of surgery, spaceflight, and their combination on other bones. As this was a multi-institutional/agency collaboration, not all tissues were available for analysis. Figure 1 details the bones available for analysis in this study, provides a representative reconstructed µCT image for each of the bones analyzed, and provides a timeline of the overall spaceflight experimental design. For each tissue, the datasets were examined for normal distributions using the Kolmogorov-Smirnov test. All data were found to have a normal distribution with the exception of trabecular humeri bone volume/tissue volume (BV/TV), trabecular number (Tb.N), and cortical humeri bone area/tissue area (B.Ar/T.Ar) as discussed below.

### Systemic effects of femoral fracture surgery/bone healing and spaceflight on trabecular and cortical bone parameters in the appendicular skeleton (tibia and humerus)

With respect to the appendicular skeleton, for this study we had access to the right tibia (n = 5/group) (on the same leg which had surgery) as well as the left humerus (n = 9–10/group).

#### Tibiae

Table 1 highlights the main tibial µCT outcomes. With respect to the trabecular compartment of the tibia, for mice remaining on earth, the bone volume fraction or bone volume/tissue volume (BV/TV) was significantly reduced by 28% in mice which had surgery (Ground + Surgery) compared to unoperated control sham mice (Ground + Sham) (p = 0.02, t-test). Interestingly, a similar decrease in tibial BV/TV was observed in spaceflight mice with or without femoral surgery (Flight + Surgery and Flight + Sham) when compared to the Ground + Sham group. In general, a reduction in BV/TV could be explained by a reduction in the trabeculae thickness (Tb.Th), a decrease in the number of trabeculae (Tb.N), and/or an increase in trabecular spacing (Tb.Sp). In these studies, 2-way ANOVA analyses showed that both gravitational condition (Ground or Spaceflight) and surgery (Sham or Surgery) significantly impacted Tb.N, Tb.Sp, and the structure model index (SMI). The Flight + Surgery mice had the greatest Tb.Sp and the lowest Tb.N, whereas Ground + Sham mice had the highest Tb.N and the lowest Tb.Sp. SMI is a measure of whether trabeculae are more “rod-like” or “plate-like” in appearance. Ideal rods are given a value of 3 and ideal plates are given a value of 0. Of note, SMI typically increases with age-related osteoporosis^14^. Consistent with this, SMI was significantly increased (29%, p = 0.02, t-test) in Flight + Sham vs. Ground + Sham mice. The Ground + Sham tibial SMI was 47% lower than that observed in the Ground + Surgery mice (p = 0.01, t-test). A similar increase in tibial SMI was also observed in Flight + Surgery compared to Ground + Sham mice.

**Table 1:** Bone parameters for the appendicular skeleton (tibia:n = 5 and humerus:n = 9–10) as measured by µCT.

Parameters	Ground		Flight	
	Sham	Surgery	Sham	Surgery
**Tibia**				
*Trabecular bone*				
BV/TV (%)	25.1(4.3)	18.1 (3.5)*	18.2 (1.5)^†^	18.9 (8.9)
Tb. Th (mm)	0.052 (0.002)	0.047 (0.005)	0.044 (0.007)^†^	0.055 (0.014)
Tb.N (mm^−1^)	**6.9 (0.4)**	**6.3 (0.5)**	**5.9 (0.8)** ^**†**^	**5.8 (0.3)** ^**†**^
Tb.Sp (mm)	**0.129 (0.010)**	**0.144 (0.012)**	**0.153 (0.017)** ^**†**^	**0.160 (0.013)**
SMI	**1.7 (0.3)**	2.5 (0.3)*	**2.2 (0.2)** ^**†**^	**2.3 (0.5)**
*Cortical bone*				
B.Ar/T.Ar (%)	71 (2)	54 (3)*	70 (5)	56 (3)*
M.Ar (mm^2^)	**0.30 (0.04)**	0.59 (0.07)*	**0.31 (0.02)**	0.59 (0.10)*
T.Ar (mm^2^)	**1.03 (0.10)**	1.28 (0.09)*	**1.03 (0.08)**	1.33 (0.14)*
B.Ar (mm^2^)	0.73 (0.08)	0.69 0.05)	0.72 (0.09)	0.74 (0.05)
**Humerus**				
*Trabecular bone*				
BV/TV (%)	13.6 (4.7)	12.6 (5.2)	14.1 (5.8)	11.3 (4.4)
Tb.Th (mm)	0.064 (0.006)	0.062 (0.006)	0.064 (0.006)	0.063 (0.007)
Tb.N (mm^−1^)	2.1 (0.6)	2.0 (0.7)	2.1 (0.7)	1.7 (0.6)
Tb.Sp (mm)	0.24 (0.04)	0.24 (0.04)	0.23 (0.05)	0.27 (0.06)
SMI	2.5 0.05)	2.6 (0.07)	2.6 (0.07)	2.6 (0.04)
*Cortical bone*				
B.Ar/T.Ar (%)	61 (2)	60 (3)	60 (5)	58 (3)
M.Ar (mm^2^)	**0.36 (0.03)**	**0.36 (0.03)**	**0.37 (0.04)**	**0.40 (0.03)** ^**†**^
T.Ar (mm^2^)	0.94 (0.07)	0.91 (0.05)	0.90 (0.06)	0.95 (0.03)
B.Ar (mm^2^)	0.58 (0.05)	0.55 (0.04)	0.53 (0.07)	0.55 (0.03)

Values are expressed as the mean ± standard deviation (SD). Bolded values indicate significant interactions were detected by 2-way ANOVA followed by Bonferroni post-hoc analyses for parametric datasets. For non-parametric datasets, Art-ANOVA was used to determine significance (no significant differences were detected). A Student’s t-test was used to detect significant differences based on (i) Surgery (e.g., Ground + Sham vs. Ground + Surgery or Flight + Sham vs. Flight + Surgery, p < 0.05, designated by *) or (ii) gravity (e.g., Ground + Sham vs. Flight + Sham or Ground + Surgery vs. Flight + Surgery, p < 0.05, designated by †). BV = Bone volume; TV = Tissue volume; Tb.Th = Trabecular thickness; Tb.N = Trabecular number; Tb.Sp = Trabecular spacing; SMI = Structure model index; B.Ar = Bone area; T.Ar = Tissue area; M.Ar = Marrow area.

The following summarizes the main tibial cortical bone parameters. Surgery, irrespective of gravity, resulted in a significant reduction in bone area/tissue area or B.Ar/T.Ar (24% reduction on earth, p = 0.0001, and a 20% reduction in spaceflight, p = 0.0004, t-test). Here, no differences were detected among the groups for B.Ar. However, T.Ar was significantly higher in mice having surgery as compared to those without surgery (24% increase on earth, p = 0.004, and a 29% increase in spaceflight, p = 0.003, t-test). Since in surgical groups no changes were observed in B.Ar, but T.Ar was significantly higher, the marrow area (M.Ar = T.Ar - B.Ar), was also significantly larger in mice having surgery as compared to those without surgery (97% increase on earth, p = 0.00001, and a 90% increase in spaceflight, p = 0.0004, t-test).

#### Humeri

**Figure 2:**
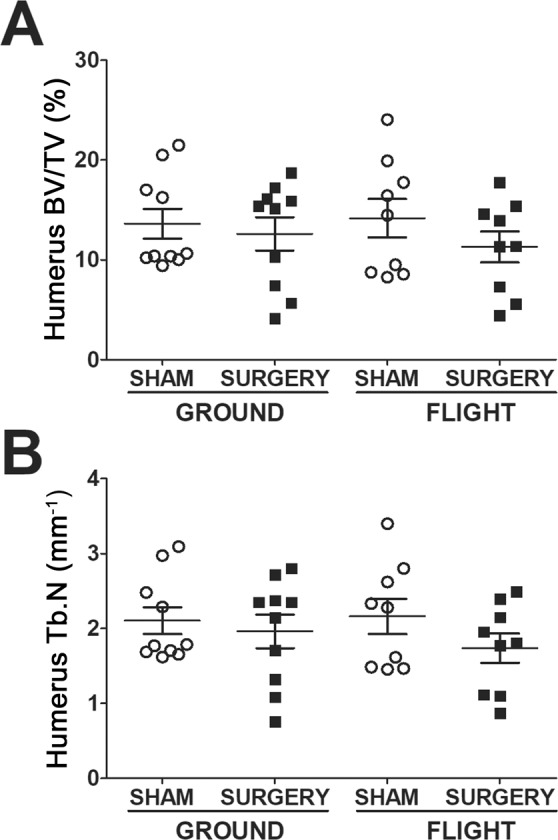
Biomodal distribution of trabecular humerus bone data. Plotting each humerus specimen shows bimodal distribution of the samples for trabecular: (**A**) Bone volume/Tissue Volume (BV/TV) and (**B**) Trabecular Number (Tb.N).

The humerus, unlike the tibia, showed no significant differences in the trabecular bone compartment as a result of microgravity for 4 weeks or the animal undergoing a major orthopaedic surgery (Table 1). However, one interesting observation from plotting out individual humeri specimens is that for both BV/TV and Tb.N, the data appear to have a bimodal distribution (Fig. 2A,B). This bimodal distribution was only identified in the humeri and was not observed in any other bones. Indeed, the Kolmogorov–Smirnov test for normality determined that only the trabecular BV/TV, Tb.N, and cortical B.Ar/T.Ar humeri datasets were non-parametric. For humeri cortical bone parameters, Flight + Surgery humeri exhibited an 11% increase in M.Ar compared to Ground + Surgery humeri (p = 0.02, t-test). With no significant differences detected in B.Ar among the 4 groups, but with an increase in M.Ar for the Flight + Surgery humeri, an increase in T.Ar would be expected. Here, there was a 4% non-significant increase in Flight + Surgery T.Ar compared to Ground + Surgery T.Ar (p = 0.07, t-test).

### Systemic effects of femoral fracture surgery/bone healing and spaceflight on bone parameters in the axial skeleton (third sternebral body, L4 vertebral body, tenth rib, calvarium, mandible, and incisor)

For this study we had access to the third sternebral body (n = 5/group), the L4 vertebral body (n = 9–10), and the tenth rib (n = 9–10). We also had access to the skull from which the calvaria (n = 9–10), mandible (n = 9–10), and incisor (n = 9–10) were assessed.

#### Sternebrae

The small sample size available for sternebrae analyses decreased our power to detect significant differences. With this caveat in mind, our µCT analysis identified some potentially interesting trends (Table 2). A non-significant 16% increase in BV/TV was seen in the sternebrae of Ground + Surgery mice compared to Ground + Sham mice (p = 0.17, t-test). The significant 11% decrease in Tb.Sp observed in Ground + Surgery mice compared to Ground + Shams (p = 0.05, t-test) explains the trending increase in BV/TV as no differences were seen in the Tb.Th nor the Tb.N. As also detailed in Table 2, no differences in BV/TV were detected between Flight + Sham and Ground + Sham sternebrae; however, there were significant decreases in both Tb.Th (10% reduction, p = 0.02, t-test) and Tb.Sp (18% reduction, p = 0.04) in the spaceflight sham animals. Additionally, there was a significant 20% reduction in BV/TV in Flight + Surgery compared to Ground + Surgery sternebrae (p = 0.04, t-test). This reduction is likely attributable to the significant 14% decrease in Tb.N (p = 0.05, t-test) and the 4% trending increase in Tb.Sp (p = 0.19, t-test).

**Table 2:** Bone parameters for the axial skeleton (sternebrae:n = 5, vertebrae:n = 9–10, and ribs:n = 9–10) as measured by µCT.

Parameters	Ground		Flight	
	Sham	Surgery	Sham	Surgery
**Sternebral body (third)**				
BV/TV (%)	9.7 (1.4)	11.3 (1.8)	9.6 (1.6)	9.0 (0.8)^†^
Tb.Th (mm)	**0.041 (0.003)**	**0.040 (0.002)**	**0.037 (0.002)** ^**†**^	**0.039 (0.001)**
Tb.N (mm^-1^)	2.4 (0.4)	2.8 (0.3)	2.6 (0.5)	2.4 (0.2)^†^
Tb.Sp (mm)	**0.231 (0.019)**	0.205 (0.011)*	**0.189 (0.031)** ^**†**^	**0.214 (0.022)**
**Vertebral body (L4)**				
BV/TV (%)	**18.7 (1.5)**	**18.5 (4.0)**	**17.4 (1.3)**	**15.9 (1.5)**
Tb.Th (mm)	0.049 (0.005)	0.048 (0.006)	0.048 (0.003)	0.045 (0.004)
Tb.N (mm^−1^)	**3.8 (0.2)**	**3.8 (0.4)**	**3.7 (0.2)**	**3.5 (0.2)**
Tb.Sp (mm)	0.202 (0.013)	0.200 (0.006)	0.203 (0.009)	0.210 (0.016)
**Rib (tenth)**				
B.Ar/T.Ar (%)	**74 (2.6)**	79 (3.5)*	**74 (2.5)**	**73 (2.1)** ^**†**^
M.Ar (mm^2^)	**0.030 (0.007)**	0.019 (0.008)*	**0.027 (0.007)**	**0.031 (0.005)** ^**†**^
T.Ar (mm^2^)	**0.115 (0.018)**	0.088 (0.024)*	**0.103 (0.019)**	**0.114 (0.013)**
B.Ar (mm^2^)	**0.085 (0.011)**	0.069 (0.017)*	**0.076 (0.013)**	**0.083 (0.008)**
Cs.Th (mm)	0.083 (0.003)	0.082 (0.006)	0.080 (0.004)	0.082 (0.002)

Values are expressed as the mean ± standard deviation (SD). Bolded values indicate significant interactions were detected by 2-way ANOVA followed by Bonferroni post-hoc analyses. A Student’s t-test was used to detect significant differences based on (i) Surgery (e.g., Ground + Sham vs. Ground + Surgery or Flight + Sham vs. Flight + Surgery, p < 0.05, designated by*) or (ii) gravity (e.g., Ground + Sham vs. Flight + Sham or Ground + Surgery vs. Flight + Surgery, p < 0.05, designated by^†^). BV = Bone volume; TV = Tissue volume; Tb.Th = Trabecular thickness; Tb.N = Trabecular number; Tb.Sp = Trabecular spacing; B.Ar = Bone area; T.Ar = Tissue area; M.Ar = Marrow area; Cs.Th = Cross-sectional thickness.

#### Vertebrae

Data for the L4 vertebral bodies are presented in Table 2. Two-way ANOVA analyses showed that spaceflight resulted in a significant reduction in BV/TV and Tb.N irrespective of surgery (p < 0.05).

#### Ribs

Both surgery and spaceflight resulted in several significant differences in the tenth rib (Table 2). A significant 7% increase in B.Ar/T.Ar was observed in Ground + Surgery ribs compared to Ground + Sham ribs (p = 0.01, t-test). Although there was a 19% reduction in B.Ar (p = 0.04, t-test) and a 25% reduction in T.Ar (p = 0.03, t-test), the smaller value of T.Ar will account for the increase in B.Ar/T.Ar. As T.Ar is the sum of the M.Ar and B.Ar, it is not surprising that a significant 37% reduction in M.Ar was also observed (p = 0.02, t-test). The other notable changes observed in the rib parameters were an 8% significant reduction in B.Ar/T.Ar (p = 0.02, t-test), a non-significant 30% increase in T.Ar, a non-significant 20% increase in B.Ar (p = 0.17, t-test), and a significant 63% increase in M.Ar (p = 0.04, t-test) in the ribs of Flight + Surgery mice compared to Ground + Surgery mice.

#### Calvaria

The parietal bone of the mouse calvarium was analyzed as shown in Fig. 1. Table 3 shows that none of the bone parameters measured were significantly different between any of the groups.

**Table 3:** Bone parameters for the axial skeleton (calvarium, mandible, and incisor) as measured by µCT.

Parameters	Ground		Flight	
	Sham	Surgery	Sham	Surgery
**Calvarium (parietal)**				
BV (mm^3^)	0.055 (0.005)	0.054 (0.003)	0.053 (0.003)	0.053 (0.005)
Width (mm)	0.164 (0.017)	0.166 (0.008)	0.156 (0.011)	0.155 (0.012)
**Mandible**				
B.Ar/T.Ar (%)	69 (2)	68 (2)	68 (2)	69 (1)
M.Ar (mm^2^)	0.61 (0.05)	0.63 (0.04)	0.63 (0.05)	0.61 (0.03)
T.Ar (mm^2^)	1.96 (0.08)	1.94 (0.02)	1.96 (0.06)	1.95 (0.05)
B.Ar (mm^2^)	1.34 (0.04)	1.31 (0.02)	1.32 (0.05)	1.33 (0.04)
CEJ-ABC (mm)	0.204 (0.036)	0.192 (0.008)	0.193 (0.025)	0.221 (0.027)
**Incisor**				
[E + D]Ar/T.Ar (%)	82 (6)	80 (9)	75 (6)^†^	82 (5)
T.Ar (mm^2^)	0.468 (0.015)	0.466 (0.005)	0.474 (0.012)	0.481 (0.015)
[E + D]Ar (mm^2^)	0.38 (0.03)	0.38 (0.03)	0.36 (0.03)	0.39 (0.04)
Pu.Ar (mm^2^)	0.083 (0.027)	0.089 (0.030)	0.116 (0.027)^†^	0.087 ± (0.023)

Values are expressed as the mean ± standard deviation (SD). Bolded values indicate significant interactions were detected by 2-way ANOVA followed by Bonferroni post-hoc analyses (no significant differences were detected). A Student’s t-test was used to detect significant differences based on (i) Surgery (e.g., Ground + Sham vs. Ground + Surgery or Flight + Sham vs. Flight + Surgery) (no significant differences were detected) or (ii) gravity (e.g., Ground + Sham vs. Flight + Sham or Ground + Surgery vs. Flight + Surgery, p < 0.05, designated by^†^). BV = Bone volume; TV = Tissue volume; B.Ar = Bone area; T.Ar = Tissue area; M.Ar = Marrow area; CEJ-ABC = Cementoenamel junction to alveolar bone crest; [E + D]Ar = [enamel + dentin] area; Pu.Ar = Dental pulp area.

#### Mandibles

As shown in Fig. 1, the mandible was analyzed after subtracting the molar and the incisor. Although no significant differences were detected between any of the groups (Table 3), there was a non-significant 15% increase in the cementoenamel junction to alveolar bone crest, or CEJ-ABC, for the Flight + Surgery compared to Flight + Sham group (p = 0.09, t-test).

#### Incisors

As illustrated in Table 3, with regard to the incisor, no significant differences were observed as a result of surgery (compared to sham mice). However, a significant 9% decrease in the percentage of enamel and dentin area ([E + D]Ar/T.Ar) was observed in Flight + Sham incisors compared to Ground + Sham incisors (p = 0.02, t-test). This appears to be a result of the 40% increase observed in dental pulp cavity or area (Pu.Ar) in the Flight + Sham incisors (p = 0.02, t-test).

## Discussion

In space, mechanical loading of the skeleton is reduced, resulting in significant atrophy of both skeletal muscles and weight-bearing bones^6–8,15,16^. In addition to reported losses in BMD, astronauts experience decreased vitamin D levels^17–19^. All of these are known independent risk factors for both falls and fracture. Importantly, predictive models^12,13^ have demonstrated that astronauts subjected to microgravity or partial gravity (Moon or Mars) will have a higher risk of wrist, femoral neck, and lumbar spine fractures than will humans on earth. During long-term spaceflights, these fractures could certainly compromise mission success and cause significant health complications for the injured astronaut. Therefore, it is important to study both the impacts of spaceflight on the skeleton and the systemic effects of fracture healing on the skeleton in microgravity environments. Consistent with previous reports of bone loss, our data demonstrated that spaceflight led to a significant 27% reduction in tibial trabecular bone fraction in unoperated Flight + Sham mice compared to unoperated Ground + Sham mice (Table 1). Similarly, surgery led to a 28% reduction in tibial trabecular bone fraction observed in mice housed on earth (same limb as injured femur, Ground + Surgery) compared to unoperated controls (Ground + Sham). Further, the Flight + Surgery mice also experienced a similar degree of tibial bone loss (25%) compared to Ground + Sham mice. Together these data suggest that both surgery to the ipsilateral femur and spaceflight result in similar, but not additive or synergistic, bone losses in the trabecular compartment of proximal tibia. This may suggest that the unloading during spaceflight and the unloading associated with altered weight-bearing after injury are responsible for this striking amount of bone loss over approximately a 4-week period of time. However, we cannot exclude that other systemic factors may be responsible for this bone loss, such as the stress response to both the spaceflight environment and injury^20^.

**Figure 3:**
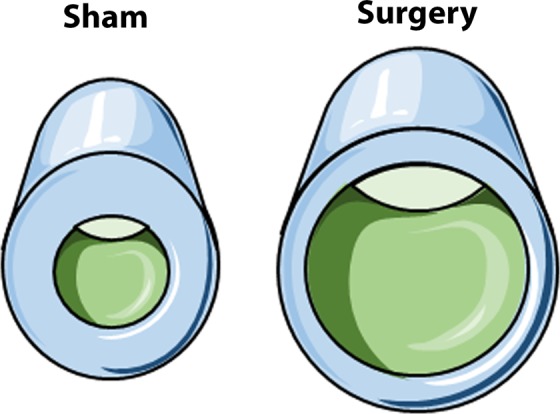
Tibia cortical bone geometry changes. The illustration shows that the cortical bone of the tibial midshaft differs in geometry between sham and surgery mice. In the cross-sectional view (excluding the shaft), the marrow area (M.Ar) is in green, the bone area (B.Ar) is in blue, and the tissue area (T.AR) is the combination of the marrow area and bone area (blue + green). Although figure dimensions are exaggerated for visual understanding, the relationship as quantified by µCT data between sham (M.Ar, B.Ar, and T.Ar) and surgery (M.Ar’, B.Ar’, and T.Ar’) areas is as follows: M.Ar’ = 2M.Ar, B.Ar’ = B.Ar, and T.Ar’ = 1.3T.Ar. These images were adapted from Servier Medical Art with permission (http://smart.servier.com/).

Another, perhaps even more impactful and intriguing finding from these studies is related to the changes observed in the cortical bone compartment of the tibia (Table 1). Here the findings were identical regardless of gravity. Thus, surgery appears to be responsible for the cortical bone changes observed. Specifically, there was a 24–29% increase in tibial cortical T.Ar when animals underwent surgery as compared to those not having surgery. This increase was not associated with a change in B.Ar; however, there was a 90–97% increase in M.Ar. Figure 3 illustrates these cortical bone geometry changes. This striking change in geometry likely leads to markedly changed bone biomechanical properties if the tissue material properties remain unchanged. However, with such a dramatic change in periosteal expansion in under 4 weeks of time, although not specifically examined here, the material properties of the remodeled bone are likely also altered. For example, with this short time period the newly formed bone is likely to be undermineralized and/or to be woven bone. Of note, since all surgical mice experienced this marked tibial geometry change within 4 weeks of surgery, whereas other long bones such as the humeri did not exhibit similar changes, the tibial geometric changes appear to be a result of either reduced weight-bearing or separate biological events that would impact only the injured leg, such as neural or vessel changes. Additionally, the injured limb itself possibly experiences localized cellular or inflammatory responses. Regardless of the mechanism, these findings may have major implications for patients suffering from femoral fractures and for the health care professionals treating these patients. While further study is needed to fully understand these findings and implications, this highlights the importance of spaceflight investigations. Specifically, because few spaceflight specimens exist, we collected and analyzed as many tissues as possible. By contrast, in our earth-based femoral bone healing studies, we typically collect and focus on the injured femur and the contralateral femur, unless a specific study design requires analysis of other tissues. Thus, identification of changes in the tibia and humerus due to surgery were only found because of this more comprehensive spaceflight study.

As mentioned above, unlike the tibia, virtually no bone parameters were altered by spaceflight or surgery in the humerus (Table 1). Spaceflight alone did not alter trabecular or cortical bone parameters in the humerus, which is consistent with a previous investigation by Lloyd *et al*.^21^, where no significant differences in bone mass were observed in 9-week-old female mice subjected to 12 days of spaceflight (NASA STS-108, using an earlier generation of the caging hardware used here). However, a very recent study by Tominari *et al*.^22^ examined the impacts of 34 days of spaceflight or artificial gravity (~1G, centrifuge hardware onboard ISS) on the trabecular and cortical bone parameters in the humerus and tibia of male C57BL/6 mice that were 9 weeks of age at the time of launch. In that study the trabecular compartment of the tibia was similar to that in our current study; however, trabecular bone in the humerus was dramatically reduced in spaceflight compared to that observed with artificial gravity. We speculate this difference in humerus data is due to differences in caging hardware and the exercise, and therefore the muscle related-loading, the humeri receive. Specifically, the NASA hardware used here (Habitat) and by Lloyd *et al*.^21^ has a larger habitable surface area (882 in^2^) and foot print (59.7 in^2^), which can co-house up to 5 mice. This results in 11.94 in^2^/mouse for the footprint and 176 in^2^/mouse for the habitable surface area (the wire mesh allows mice to climb and access much of the 6 surfaces), and is well above the ≥8 in^2^ recommendation for mouse housing listed in the *Guide for the Care and Use of Laboratory Animals*^23^.

In contrast, the Japan Aerospace Exploration Agency (JAXA) habitat cage units (HCUs) house mice singly and are significantly smaller than the NASA Habitat as shown in their video footage, where mouse movements are limited^22^. Although permission has not yet been received from NASA to release videos of the mice used in these studies, the videos were examined by several members of the team including RH and MAK. The NASA videos show two routine behaviors of active mice in spaceflight. One is “floating,” and just like astronauts who use their arms to pull themselves from one end of the ISS to the other, floating mice use their forelimbs to push off or pull themselves from one end of the wire mesh cage to the other. The second behavior is running laps. Both of these behaviors would provide loading to the humerus, which does not appear to happen in the JAXA HCUs^24^. The two separate behaviors, running laps and floating, may also explain the biomodal distribution of the spaceflight humeri data presented in Fig. 2. For example, perhaps the mice with the higher BV/TV measurements were the mice running laps and were mechanically loading their humeri, whereas the cohort with lower BV/TV measurements may be the less mobile, floating mice. With similar logic, perhaps the humeri bone parameters of earth-housed mice had a bimodal distribution based on animal activity. These possibilities require further investigation. Another difference between the studies is co-housing of mice in our studies versus singly housed mice in the JAXA studies by Tominari *et al*.^22^ As mice are social animals, singly housing mice may lead to chronically elevated levels of stress, which could also impact bone parameters^25–27^.

In addition to the tibia and humerus, the sternebra, L4 vertebra, and tenth rib of the axial skeleton were examined. Most previous reports on the effects of microgravity on bone physiology examined weight-bearing bones; to our knowledge this is the first report looking at the sternbrae and ribs. With regard to the sternebrae (Table 2), perhaps the most intriguing observation is that the Ground + Surgery mice exhibited a >15% increase in BV/TV compared to all other groups. Although, this difference was only significant compared to Flight + Surgery mice, with a larger sample size, this difference might reach significance.

As shown in Table 2, for the L4 vertebrae there was a significant reduction in BV/TV in spaceflight mice compared to mice housed on the earth (2-way ANOVA). These trends were similar to that documented by others for spaceflight investigation of lumbar vertebrae^28,29^. As also detailed in Table 2, the ribs showed significant reductions in the B.Ar, T.Ar, and M.Ar in mice having surgery and housed on earth compared to sham-operated controls also housed on earth (Ground + Surgery vs. Ground + Sham); as breathing is a major mechanical stimulator of ribs, this likely reflects more shallow breathing post-surgery^30^. Interestingly, spaceflight also resulted in a significant reduction in rib B.Ar in unoperated control animals (Flight + Sham vs. Ground + Sham), which may be explained by the reduction in rib cage expansion observed during breathing in astronauts^31^.

Although several studies have been conducted on the effects of spaceflight on the soft tissue present in the skull, including reports of spaceflight-associated decreases in frontal and temporal gray matter volumes, increased somatosensory cortex, brain displacement within the skull, and ventricular volume expansion^32–35^, little is known on the effects of bone growth and remodeling. Previously, murine calvaria volume and thickness were reported to increase during the 15-day NASA Shuttle mission STS-131^36^; however, when the same group repeated the spaceflight study with a longer mission duration (30 days), they observed no significant changes in murine calvaria bone thickness and volume when compared to ground controls^37^. Here we also examined a longer mission duration (~4 weeks) and observed no significant changes in murine calvaria bone parameters via µCT (Table 3). We also did not see an increase in B.Ar in the mandible even though bone volume (region of interest *B.Ar) was reported to increase during STS-135’s 30-day spaceflight mission^38^. We did, however, note a non-significant 15% increase in the cementoenamel junction to alveolar bone crest distance or CEJ-ABC for the Flight + Surgery compared to the Flight + Sham group, suggesting the mandible may experience bone growth. We also observed a non-significant 5% decrease in the CEJ-ABC between Flight + Sham and Ground + Sham mice, which is consistent with the reports of Gosh *et al*.^39^. Interestingly, we report a decrease in enamel and dentin T.Ar in Flight + Sham incisors compared to Ground + Sham incisors due to a 40% increase in dental pulp cavity or area (Pu.Ar) in the Flight + Sham incisors. These data indicate that normal dental growth of the incisor may be impaired in spaceflight mice. Of note, the increase in dental pulp area seems to parallel the tibial marrow expansion.

The opportunities to conduct spaceflight studies are limited, and the resources available for these studies are minimal. This results in small sample sizes, restricted opportunities to reproduce findings, and differences in study designs such as age, sex, strain, and general health (wild-type vs. genetically altered or healthy vs. injured mice etc.). Further, different countries and institutions have access to different animal hardware/cages, which can also impact results. Here we compare skeletal findings in our unoperated “sham” mice with what has been shown by others, but also report novel findings that surgery has some systemic impacts on bones, some of which appear to be independent of gravity, whereas others appear to be dependent on gravity. Thus, additional studies will be required to more fully understand the impacts of spaceflight and surgery on the skeleton. That said, the studies presented here highlight the importance of continuing to complete spaceflight investigations as well as the importance of examining as many tissues as possible, not just the primary tissue impacted by whatever intervention is being investigated.

## Conclusions

In conclusion, the studies described here illustrate the systemic effects of fracture healing, of spaceflight, and of the two combined, on several bones within the appendicular and axial skeleton. Two of the most important findings in this study were changes observed in the tibia. The first was that both spaceflight and surgery resulted in a significant, ~25% reduction in trabecular BV/TV in the tibia on the injured limb. The second was that surgery, irrespective of gravity, resulted in dramatic cortical bone changes in the tibia on the surgical limb, including significant periosteal and marrow expansion. These findings are not only relevant to astronauts but also important to fracture patients on earth as well as to their caregivers.

## Materials and Methods

### Animals

Seven-week-old male C57BL/6 J mice were purchased from Jackson Laboratories (Bar Harbor, ME). Mice were allo-reared, or cage-mated from weaning, in cohorts of 15 mice in N40 mouse cages (Ancare, Bellmore, NY). A full description of the acclimation process and experimental housing conditions was previously published^40–42^. In brief, once mice arrived at the Kennedy Space Center (KSC), they were placed in N40 cages containing a raised wire floor (3 openings/inch, Ancare) and were provided modified lixit water bottles and NASA Nutrient-upgraded Rodent Food Bar (NuRFB) to acclimate mice to spaceflight-like hardware/food. Mice were ear punched for identification purposes and weighed twice/week to determine whether they had adapted to utilization of the special water lixit and the NuRFB. Mice were maintained on a 12-hour light/dark cycle. Ambient temperature for all animals was maintained at 24–25 °C. All experiments were performed in accordance with the NIH Guide for the Care and Use of Laboratory Animals, and followed approved experimental protocols (NASA Animal Care and Use Committees, #FLT-15-101/NAS-15-105).

### Groups

These data are part of a larger experiment that addresses fracture healing in space aboard the ISS as part of NASA’s Rodent Research 4 mission. This manuscript represents data from spaceflight (Flight) and ground (Ground) mice that underwent segmental defect (Surgery) or sham surgery (Sham). It should be noted that ground controls were asynchronous by 5 days so that all conditions aboard the ISS could be replicated on earth for the ground controls. This includes environmental conditions such as cage temperature, food and water changes, and the timing from euthanasia to placement of dissected specimens into the cold stowage/freezers.

### Surgery

Two weeks after arrival at KSC and 4 days prior to launch, entire cages of mice were randomized into the following 4 groups: (1) Sham surgery housed on the earth (Ground + Sham); (2) Segmental bone defect surgery housed on the earth (Ground + Surgery); (3) Sham surgery housed on the ISS (Flight + Sham); or (4) Segmental bone defect surgery housed on the ISS (Flight + Surgery). The surgical protocol has been described previously^40,41^. In brief, mice were anesthetized with Ketamine-Xylazine (125–20 mg/kg), and the right leg was shaved and scrubbed three times with ethanol and Betadine to ensure the limb was sterile. Next, a 1 cm lateral incision was made over the right femoral midshaft. After the initial incision, the knee was then flexed, and a 27-gauge needle was manually inserted between the condyles of the femur and threaded retrograde into the intramedullary canal. The needle was then partially removed, and a sterile Dremel rotary cutting tool (Dremel Inc., Racine, WI) was used to remove a 2 mm intercalary segment from the femoral diaphysis. Next, a synthetic graft composed of poly(propylene fumarate)/tricalcium phosphate was inserted into the defect site to maintain the defect size^43^. The needle was then advanced through the synthetic graft and using a twisting motion bore through the greater trochanter. The exposed needle tip was then bent back on itself, and the needle was pulled anterograde to stabilize the femur and defect. The opposite end of the needle was cut as close to the distal femur as possible. Next, a saline-soaked collagen sponge (RCM6 Resorbable Collagen Membrane, ACE, Brockton, MA) was wrapped around the synthetic scaffold and sutured into place. The muscle was sutured closed and then the skin was closed using wound clips (7 mm, Braintree Scientific, Braintree, MA). Mice were monitored until they recovered from the anesthetic. After recovery, mice were returned to their original cages, and K3392 Rest Stops or resting boards (Bio-Serve, Flemington, NJ) were added for the first 2 days of recovery before the 10 healthiest mice/group (determined by NASA veterinarians in collaboration with MAK and PJC) were transferred into spaceflight hardware 2 days prior to launch (NASA Rodent Transporters which house the mice while they are on SpaceX and NASA Rodent Habitats which house mice while they are on the ISS)^42^.

### Sample collection

Mice were approximately 9 weeks old at launch (SpaceX CRS-10, February 19, 2017) and approximately 13 weeks old at euthanasia. Specifically, mice were euthanized between 24 and 28 days post-launch as astronauts could only euthanize and dissect 8 mice/day. Mice were euthanized by injection of ketamine/xylazine (150/45 mg/kg), a closed chest cardiac puncture with blood withdraw, followed by a cervical dislocation. For five mice in each group, the right hindlimb was removed at the hip and placed in 10% neutral buffered formalin or NBF (transferred to 4 °C within ~4–6 hours). These specimens remained at 4 °C in 10% NBF until they were returned to Indiana University School of Medicine approximately 2 weeks after euthanasia. Then the samples were washed with ice cold phosphate buffered saline (PBS), transferred into ice cold 70% ethanol, and then stored at 4 °C until they underwent µCT scanning as described below. The remaining carcass was wrapped in aluminum foil and transferred to the −95 °C cold storage unit aboard the ISS (on Earth transferred to the −80 °C freezer). For the other 5 mice in each group, the carcass remained intact, and the whole carcass was wrapped in aluminum foil and frozen. Carcasses remained at −80 °C or below while on the ISS or while maintained at Kennedy Space Center, through shipping to the US Army Center for Environmental Health Research at Fort Detrick, MD approximately 2 weeks after euthanasia. The mice were then partially thawed (placed on ice blankets) for ~15 minutes, tissue dissection was completed for each carcass (~45 minutes total time spent outside of the −80 °C freezer) and the bones investigated here were immediately snap frozen aside from the humerus. For the humerus the whole forelimb was placed in 10% NBF for 72 hours, washed with ice cold PBS, transferred into ice cold 70% ethanol, shipped to Indiana University School of Medicine at 4 °C and then stored at 4 °C until they underwent µCT scanning. The frozen bone specimens were also shipped to Indiana University School of Medicine, but on dry ice, and were then stored at −80 °C until they were removed for specific dissection as detailed below.

Here we are reporting on the systemic impacts of spaceflight and segmental bone defect surgery on the axial and appendicular skeleton; fracture healing results will be published in a separate report.

### Micro-computed tomography

Tibias were imaged using a desktop SCANCO µCT35 imaging system (SCANCO Medical, Brüttisellen, Switzerland) with all scans obtained at 55 kV using a 12 μm voxel size. For trabecular analyses of the tibias (n = 5), the region of interest (ROI) is also the tissue volume (TV) and was defined as the region 0.25 mm distal of the proximal growth plate and extended an additional 0.5 mm proximally. Variables recorded include: bone volume/tissue volume (BV/TV), trabecular thickness (Tb.Th), trabecular number (Tb.N), trabecular spacing (Tb.Sp), and structure model index (SMI). With respect to the cortical analysis of the tibia, a 1 mm ROI was obtained from a region that was 0.25 mm proximal from the tibiofibular junction. The area contained within the periosteal surface is the cortical TV. Initial variables recorded were BV and cortical thickness (Ct.Th). BV was converted to bone area (B.Ar) by dividing by the height of the analyzed bone segment (1 mm). Marrow area (M.Ar) and tissue area (T.Ar) were calculated by the equation for the area of a cylinder: B.Ar = π*(total radius^2^ − marrow radius^2^). By substituting total radius = Ct.Th + marrow radius and solving for marrow radius, we obtained the following equation: marrow radius = ((B.Ar/π) − Ct.Th^2^)/(2*Ct.Th). Next, M.Ar was calculated as π*marrow radius^2^, and T.Ar was calculated as B.Ar + M.Ar.

For all frozen bones analyzed in this study, samples were briefly thawed, surrounding soft tissue was removed, and the bone of interest was isolated and fixed for 72 hours in 10% NBF, washed with ice cold PBS, and then stored in ice cold 70% ethanol at 4 °C. A desktop SkyScan 1172 µCT imaging system (SkyScan, Kontich, Germany) was used to image humeri, calvariae, mandibles, ribs, sternebra, and vertebrae. Scans were obtained at 60 kV using a 5.9 μm voxel size for the humeri, calvaria, mandibles, sternebra, and vertebrae. The rib scans were obtained at 60 kV using a 9.8 μm voxel size. Images were reconstructed (NRecon v.1.7.3) and analysis was carried out on Skyscan software (Dataviewer, CTAn, Kontich, Belgium).

With regard to trabecular analyses of the humeri (n = 9–10), the ROI started at 0.5 mm distal from the proximal growth plate and extended an additional 0.5 mm distally. 3D analyses were completed to obtain BV/TV, Tb.Th, Tb.N, Tb.Sp, and SMI. For the cortical analyses of the humeri, the ROI was set at 0.5 mm proximal from the midshaft and extended an additional 0.5 mm proximally, to avoid the deltoid tuberosity. 2D analyses were completed to obtain B.Ar, T.Ar, B.Ar/T.Ar, M.Ar, and Ct.Th.

With regard to trabecular analyses of the sternum (n = 5) and vertebrae (n = 9–10), ROIs were obtained from 1 mm tall segments centered at the third sternebral body or the L4 vertebral body. 3D analyses were conducted and recorded variables include BV/TV, Tb.Th, Tb.N, and Tb.Sp.

For the ribs (n = 9–10), a 0.5 mm ROI was analyzed at the midshaft of the tenth rib. Cortical rib parameters recorded include B.Ar, T.Ar, B.Ar/T.Ar, M.Ar, and cross-sectional thickness (Cs.Th).

With respect to calvaria (n = 9–10), the ROI was a 100 pixel^3^ volume centered at the parietal eminence. 3D analyses were completed and TV and BV were obtained. BV/TV and marrow volume (MV = TV–BV) were also calculated. Additionally, calvarial width or thickness was obtained by collecting 3 width measurements from 3 random images.

For the mandible (n = 9–10) the ROI was a single coronal slice taken through the middle of the posterior root of the first molar. The molar was then subtracted from the ROI prior to performing a 2D analysis on the mandible with the incisor. A separate 2D analysis was also completed on just the incisor. Mandible variables included T.Ar, B.Ar, and M.Ar. The latter was calculated as follows: M.Ar = T.Ar − B.Ar. Of note, the mandible values were obtained by subtracting the equivalent incisor values (T.Ar; dentin + enamel area [D + E]Ar; pulp area or Pu.Ar = T.Ar − [D + E]Ar). For both the mandible and incisor ROIs the shrink-wrap function was used to ensure accurate T.Ar measurements. Additionally, the lingual cementum–enamel to alveolar bone crest distance (CEJ–ABC) was acquired by measuring the distance from the cementum edge on the lingual tooth surface to the alveolar bone apex.

It should be noted that one Flight + Surgery mouse was euthanized in spaceflight ~1 week after launch based on veterinarian recommendation due to inactivity of the mouse compared to cage-mates. This resulted in n = 9 in the Flight + Surgery group as compared to n = 10 in the other 3 groups where applicable.

### Statistics

All data were tested for normality using the Kolmogorov–Smirnov test. Parametric data between sham and surgery, and, ground and flight were analyzed by two-way ANOVAs followed by Tukey post-hoc analyses. For some analyses, a Student’s t-test was also used. A significance threshold was set at α = 0.05. Statistical analyses were determined using Prism v5.0. Non-parametric data was analyzed using Art-ANOVA^44^ and Microsoft Excel office 2010 (Microsoft corporation, Redmond, WA). Graphs were generated using Prism v5.0 (GraphPad, San Diego, CA) and figures were generated using Adobe Photoshop (Adobe, San Jose, CA).

## Data Availability

The data that support the findings of this study are available from the corresponding author upon reasonable request.
